# A highly conserved gene island of three genes on chromosome 3B of hexaploid wheat: diverse gene function and genomic structure maintained in a tightly linked block

**DOI:** 10.1186/1471-2229-10-98

**Published:** 2010-05-27

**Authors:** James Breen, Thomas Wicker, Xiuying Kong, Juncheng Zhang, Wujun Ma, Etienne Paux, Catherine Feuillet, Rudi Appels, Matthew Bellgard

**Affiliations:** 1 Centre for Comparative Genomics (CCG), Murdoch University, South Street, Perth 6150, Australia; 2 Molecular Plant Breeding Co-operative Research Centre (MPBCRC) Murdoch University, South Street, Perth 6150, Australia; 3 Institute of Plant Biology, University Zurich, Zollikerstrasse 107, Zurich, CH-8008 Switzerland; 4 Key Laboratory of Crop Germplasm Resources and Utilization, MOA/Institute of Crop Sciences, CAAS/The Key Facility for Crop Gene Resources and Genetic Improvement, Beijing 100081, China; 5 State Agricultural Biotechnology Centre (SABC), Murdoch University, South Street, Perth 6150, Australia; 6 Department of Agriculture and Food, Western Australia (DAFWA), 3 Baron Hay Court, Perth, 6151 Australia; 7 UMR 1095 Génétique, Diversité et Ecophysiologie des Céréales, INRA Site de Crouël, 63100 Clermont-ferrand, France

## Abstract

**Background:**

The complexity of the wheat genome has resulted from waves of retrotransposable element insertions. Gene deletions and disruptions generated by the fast replacement of repetitive elements in wheat have resulted in disruption of colinearity at a micro (sub-megabase) level among the cereals. In view of genomic changes that are possible within a given time span, conservation of genes between species tends to imply an important functional or regional constraint that does not permit a change in genomic structure. The *ctg1034 *contig completed in this paper was initially studied because it was assigned to the *Sr2 *resistance locus region, but detailed mapping studies subsequently assigned it to the long arm of 3B and revealed its unusual features.

**Results:**

BAC shotgun sequencing of the hexaploid wheat (*Triticum aestivum *cv. Chinese Spring) genome has been used to assemble a group of 15 wheat BACs from the chromosome 3B physical map FPC contig *ctg1034 *into a 783,553 bp genomic sequence. This *ctg1034 *sequence was annotated for biological features such as genes and transposable elements. A three-gene island was identified among >80% repetitive DNA sequence. Using bioinformatics analysis there were no observable similarity in their gene functions. The *ctg1034 *gene island also displayed complete conservation of gene order and orientation with syntenic gene islands found in publicly available genome sequences of *Brachypodium distachyon*, *Oryza sativa*, *Sorghum bicolor *and *Zea mays*, even though the intergenic space and introns were divergent.

**Conclusion:**

We propose that *ctg1034 *is located within the heterochromatic C-band region of deletion bin 3BL7 based on the identification of heterochromatic tandem repeats and presence of significant matches to chromodomain-containing *gypsy *LTR retrotransposable elements. We also speculate that this location, among other highly repetitive sequences, may account for the relative stability in gene order and orientation within the gene island.

Sequence data from this article have been deposited with the GenBank Data Libraries under accession no. GQ422824

## Background

Wheat (*Triticum aestivum*) is one of the major food crops in the world, providing 20% of the total food calories and protein in human nutrition [1] but its large genome size (~16000 Mb) and complexity (~80% repetitive sequences) has hindered genome sequencing studies [2]. To date, the largest sequenced, assembled and annotated genomic sequence from BAC clones from Triticeae genomes is 439 kb [3]. The recent publication of the physical map of the largest wheat chromosome 3B [4], along with other mapping projects initiated within the international wheat genome sequencing consortium (IWGSC), has established a platform for more extensive sequencing studies.

The high complexity of the wheat genome produced by such factors as retrotransposable element insertion [5] and polyploidy, has complicated comparisons between the genomes of closely related species. Gene deletions and disruptions generated by the fast replacement of repetitive elements in wheat [6-8], have resulted in loss of colinearity at a micro (sub-megabase) level [9-12]. In view of genomic changes that are possible within a given time span, conservation of gene structure, order and orientation between species tends to imply an important functional or regional constraint that does not permit a change in genomic structure [13]. The *ctg1034 *sequence characterized in the present study is a good example of such high conservation of structure.

The availability of finished, whole genome sequences for rice (*Oryza sativa*) [14] and *Sorghum bicolor *[15], as well as extensive sequencing of the maize (*Zea mays*; http://www.maizesequence.org) and Brachypodium (*Brachypodium distachyon *http://www.brachypodium.org) genomes, has provided a basis for a detailed analysis of colinearity. Due to a lack of whole genome sequence data for wheat, micro-colinearity has been confirmed on individual sequenced genetic loci of agronomically important genes [16,17] but little work has been carried out to identify any detailed relationships in regions of low gene density.

Gene islands exist in grass genomes [18-21] and gene-densities of over one gene every 20 kb have been reported [7,22]. On wheat chromosome 3B, Charles et al. [5] carried out random BAC sequencing to identify transposable element distribution and annotated the very few active genes within distal regions of the long-arm of the chromosome. Only one gene island (containing two active genes) was identified out of the 10 annotated BAC clones.

In this study we report on the sequencing of a group of 15 BACs located within FPC *ctg1034 *from the hexaploid wheat chromosome 3B physical map [4], and their assembly into a 783,553 bp genomic sequence. This large genomic sequence is among the first reported for the long arm of chromosome 3B and is also one of the largest and most complete genomic sequences described for wheat. The 783,553 bp genomic sequence was initially studied because it was assigned to the *Sr2 *resistance locus region, however detailed mapping studies subsequently assigned it to the long arm of 3B and revealed its unusual features. Annotation of *ctg1034 *through bioinformatics analysis identified a conserved gene island containing three genes of different gene function. Comparative analysis with five other cereal genomes revealed the highly conserved nature of the gene island despite no apparent co-expression or other shared functional attributes being identified.

## Results

### *Ctg1034 *sequence assembly and chromosome 3B mapping

**Figure 1:**
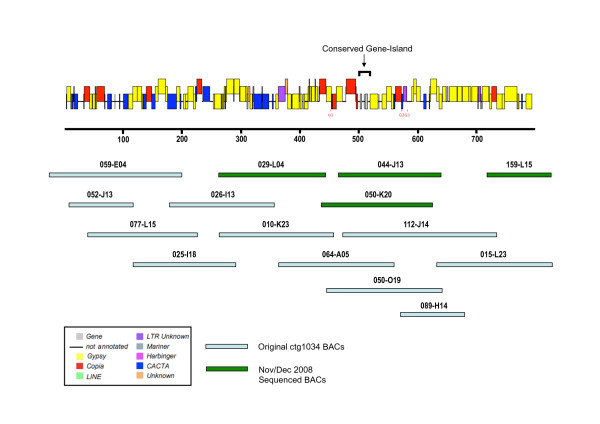
**Genome sequence annotation of *ctg1034 *showing the wheat BAC clones that were used in the assembly of the genomic sequence**. The retrotransposable elements have been color coded as indicated in the insert.

The sequencing of chromosome 3B-*ctg1034 *from *Triticum aestivum *cv. Chinese Spring was initially carried out using a BAC-by-BAC shotgun method at a 6× sequencing coverage. Twelve BACs (026-I13, 025-I18, 077-L15, 059-E04, 052-J13, 010-K23, 111-P10, 064-A05, 050-O19, 112-J14, 089-H14 and 015-L23) were originally selected from the chromosome 3B physical map generated by FPC mapping [4] (Figure 1). Gap closure was carried out by identifying sequence read pairs and designing primers to extend the genomic sequence. A second set of four BAC clones (029-L04, 044-J13, 159-L15 and 050-K20) was sequenced at 10× coverage in an attempt to close all remaining genomic sequence gaps (Figure 1). The sixteen wheat BACs were assembled using the Phred/Phrap sequence assembly package [23]. BAC contigs were ordered and assembled into a scaffold sequence by using target site duplications (TSDs) located at the ends of transposable elements (T. Wicker et al manuscript *in preparation*). TSDs associated with a particular transposable element allowed this element to be annotated even if nested insertions had occurred within it to complicate its internal structure.

**Figure 2:**
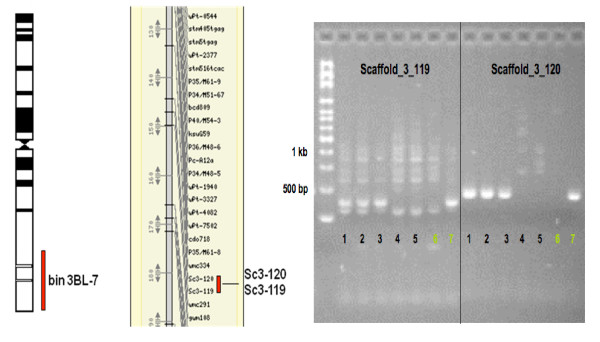
**Location of *ctg1034***. A (left panel). Genetic mapping using two ISBP markers, Sc3-119 and Sc3-120 on the Cranbrook × Halberd population [26] demonstrated a co-location with markers assigned to bin3BL-7 on the long arm location on 3B [72]. The genetic map shows a small part of the map (complete map, Cran*Hal 3B Feb09, is available at http://ccg.murdoch.edu.au/cmap/ccg-live/). B (right panel). The bin 3BL-7 region of chromosome 3B is characterized by the SSRs Xgwm299, Xgwm2152, Xgwm547, Xgwm247, Xgwm340, Xgwm181, Xfwm4, Xcfa2170, Xbarc84, Xpsr170, XksuG62 [72] and the two ISBP markers (Sc3-119 and Sc3-120) could be assigned to this deletion bin. Lane 1-8 on the electrophoresis gel on the left-hand panel of the figure indicates the analysis of Sc3-119 and Sc3-120 with the deletion bins of chromosome 3B (lane 1: 3BS-8, lane 2: 3BS-9, lane 3: 3BS-1, lane 4: 3BL-10, lane 5: 3BL-7, lane 6: Halberd and lane 7: Cranbrook).

The BAC contigs sequenced in this study were initially linked to BACs located in the region of the *Sr2 *locus (Mago et al. *in preparation*) of chromosome 3BS and the contig was of interest because of the identification of a defensin gene within the gene island. However, when no overlap could be found within assembled contigs, markers were tested to re-examine the chromosome location of the BACs. Two insertion site based polymorphism (ISBP) markers [7,24,25] were identified in the sequence and used to screen the Cranbrook × Halberd mapping population [26] to find the chromosomal location of *ctg1034 *on chromosome 3B. Figure 2(A) shows the genetic mapping analysis results on the Cranbrook × Halberd mapping population which confirmed earlier indications from studies using a French (INRA) mapping population that contig 1034 was co-located with molecular markers previously assigned to bin 3BL-7, on the long arm of 3B. Confirmation of the location in bin 3BL7 is provided in Figure 2B using the same ISBP markers used for the genetic mapping. The ISBP Sc3-119 marker primers (predicted PCR band size of 158 bp) showed multiple PCR bands with the band of the expected size missing from lanes 4 and 5. For ISBP Sc3-120, the PCR showed a single band of the expected size (190 bp) and this band was missing in lanes 4 and 5. Since lanes 3 and 4 are deletion lines 3BL10 and 3BL7 the analysis indicated that the locus containing the ISBP is located on 3BL7 [4].

### Transposable element annotation of *ctg1034*

Transposable elements (TEs) were annotated in this study using BLASTN and BLASTX [27] sequence homology searches against the non-redundant nucleotide (e-value < 1e^-30^) and protein (e-value < 1e^-10^) sets of the Triticeae repetitive element (TREP) database. Alignments to TEs located within *ctg1034 *were confirmed using the DOTTER dot-matrix program [28] and were used to identify target site duplications (TSDs) at either end of the element. Table 1 lists the TE families that are distributed within *ctg1034 *and their sequence proportions compared to the rest of the assembled contig.

**Table 1:** Transposable element Annotation of Chromosome 3B *ctg1034*

Class	Order	Family	Code	Elements	Complete TSD	Nested	Length (bp)	% TEs	% Contig
Class I (Retrotransposable elements)									
	LINE	Unknown	RIX	1		1	2962	0.46%	0.38%
	LTR	Copia	RLC	9	9	1	93966	14.66%	11.99%
		Gypsy	RLG	55	39	19	401485	62.64%	51.24%
		Unknown	RLX	6	3	2	29915	4.67%	3.82%
Total				**71**	**51**	**23**	**528328**	**82.42%**	**67.43%**
Class II (DNA Transposons)	TIR	CACTA	DTC	9	5	5	100864	15.74%	12.87%
		Harbinger	DTH	1			1838	0.29%	0.23%
		Mutator	DTM	1			802	0.13%	0.10%
		Tc1- mariner	DTT	1			693	0.11%	0.09%
	Unknown		DXX	1			730	0.11%	0.09%
Total				**13**	**5**	**5**	**104927**	**16.37%**	**13.39%**
Unknown			XXX	2			7733	1.21%	0.99%
Total Transposable elements				**86**	**56**	**28**	**640988**		**81.81%**

Overall, TEs made up 81.8% of the entire *ctg1034 *chromosome 3B sequence. The repetitive element content was dominated by retrotransposable elements (class I TEs, 82.42% of all TEs and 67.43% of the entire contig), considered to transpose through the action of a RNA intermediate. This was compared to the DNA transposons (class II), which comprised only 16.37% of all TEs (13.39% of the total contig). The *gypsy *family of long terminal repeat (LTR) retrotransposable elements were by far the most abundant type of class I-TE, with 71 out of the 86 transposons found (51 of which were complete with intact TSDs) and occupying more than half the total sequence of *ctg1034 *(51.24%).

Two *gypsy *elements, *Romani *located at 698,865 - 709732 bp and *Latidu *located at 510,556 - 523623 bp in *ctg1034*, were also found to contain significant matches to chromodomains, which are located in the C-terminal region of the integrase (IN) protein domain of the *gypsy *internal polyprotein (*gag-pol*) domain [29]. A profile HMM [30] constructed using consensus sequences of *reverse transcriptase *(RT) and IN domains in [31] was used to identify potential chromodomain matches (e-values of 5.7e^-307 ^and 1.5e^-303 ^for *Romani *and *Latidu *respectively). Chromodomains were originally identified in the centromeric heterochromatin proteins of *Drosophila *[32,33] and have been argued to target heterochromatin by recognizing histone modifications [31]. The internal regions of both the *Romani *and *Latidu *LTR *gypsy *elements were also searched using BLASTX against the characterised internal domains of the *gypsy *mobile genetic element database (GyDB) [34]. Both elements contained significant matches (evalue of 1e^-149^) to the integrase domain of the *O.sativa *LTR *gypsy *element *Retrosat-2*.

The *CACTA *DNA transposons and *copia *LTR families were also well represented with 12.87% and 11.99% of the total *ctg1034 *sequence respectively. The *CACTA *DNA transposons had a high proportion of elements (5 out of the 9 elements found) that contained nested insertions. *Gypsy *LTR retrotransposons had a slightly lower proportion of nested elements (19 out of 55). The *copia *LTR elements were found to be the most intact with only one element containing a nested insertion and all identified to be complete with TSDs.

Tandem repeats considered to be located within heterochromatic regions in Triticeae genomes were also assayed to identify matches to *ctg1034*. Two matches were genome specific' *Aegilops squarrosa *repetitive DNA sequence (GenBank accession: D30736) [35] and the other being an *Aegilops tauschii *so-called centromeric-specific tandem repeat from clone 6C6-3 (Genbank accession: AY249982) [36]. The well-studied D30736 repeat [35] is from the *Afa1 *family of tandem repeats (based on the restriction enzyme site) and is made up of 240 bp units, only one of which was located in the *ctg1034 *sequence between 72,048 and 72,279 bp (89% nucleotide identity over 233 bp). The AY249982 repeat was almost a perfect match (92% nucleotide identity over 1018 bp) between 40,882 and 41,898 bp. Both these representatives of tandem repeat families were located within TEs, with the D30736 repetitive DNA sequence found within the CACTA DNA transposon *Conan *and the AY249982 tandem repeat found within the *gypsy *LTR retrotransposon *Cereba*.

### Gene content annotation of *ctg1034*

The 783,553 bp *ctg1034 *sequence was masked for repetitive elements with the repeat masking program Repeatmasker http://www.repeatmasker.org run using the triticeae repetitive element (TREP) database http://wheat.pw.usda.gov/ITMI/Repeats. The masked sequence was then passed through gene prediction programs FGENESH http://linux1.softberry.com/berry.phtml and GlimmerHMM [37]. Predicted gene models from both programs were screened against the Michigan State University (MSU) *O. sativa *annotation project's http://rice.plantbiology.msu.edu/ protein database and NCBI non-redundant amino-acid database using BLASTP to identify non-redundant gene matches. A database of 11,902 full-length wheat cDNA sequences from the KOMUGI Wheat Genetic Resource http://www.shigen.nig.ac.jp/wheat/komugi/ was also screened to identify any direct matches to wheat cDNAs. All annotated genes followed the standard GT-AG eukaryotic intron splice site model.

Four gene models of significant homology to characterised protein matches in NCBI non-redundant amino acid and MSU rice protein database were all found within a 16 kb window between 491,000-507,000 bp of the *ctg1034 *contig. The first gene model found at the very beginning of the 16 kb region was identified to be a fragment (28% coverage) of the MSU rice gene hit, LOC_Os05 g35160 (Clathrin assembly protein) with no significant wheat EST or cDNA match and was not considered further. The other three gene models were annotated as gene-coding sequences and all had hits to the NCBI non-redundant protein database. All three genes were annotated using BLASTX alignments to the MSU rice protein annotations [38] and their intron-exon structure was confirmed using both a wheat subset of Expressed Sequence Tags (ESTs) from NCBI and rice full-length cDNA information [39]. The overall gene-density of the *ctg1034 *sequence was 1 gene per 261,184 bp.

**Figure 3:**
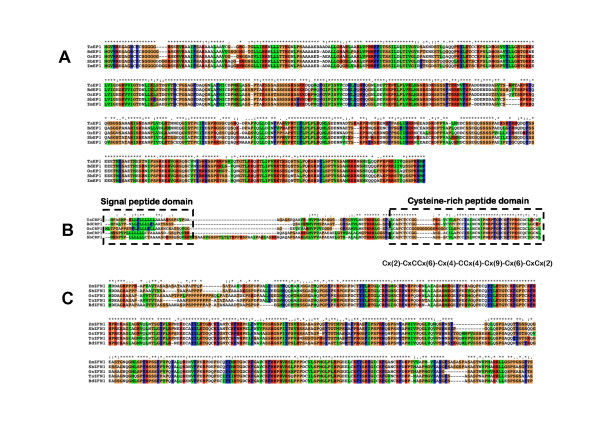
**Multiple sequence alignment of three collinear gene island genes (*TaEP1 *(A), *TaCRP1 *(B) and *TaZFN1*(C)) using CLUSTALX **[78]. Each of the wheat, Brachypodium and maize genes were annotated in this study, while the *S. bicolor *(v1.0 annotations from http://www.phytozome.net/sorghum) and rice annotations [38]. Annotated on the *TaCRP1 *alignment is the signal peptide and cysteine-rich domain structures outlined in [40]. Colors found on the figure are determined by the CLUSTAL color scheme, which is based on amino acid types.

The first of the three genes, named *TaEP1*, was identified to be 2,489 bp in length and encoded a predicted protein of 551 amino acids over 2 exons (Table 2). The gene has a high similarity at the amino acid level (85% identity) to an expressed protein in rice (LOC_Os01 g68830) as well as a catalytic domain-containing protein in Arabidopsis (*Arabidopsis thaliana*; At3 g07210) (49% identity). The gene was supported by multiple wheat full-length ESTs (CJ799192, BE500656, BG263254, BQ170023) all with a greater than 95% nucleotide identity. The second gene (*TaCRP1*) was quite small at only 436 bp in length and encoded a predicted protein of 118 amino acids over 2 exons (Table 2). The gene was well supported by multiple full-length ESTs (CA636856, BQ806045, CA702257 and CA707955), which all had a greater than 95% nucleotide identity. The predicted protein of the gene was found to have homology (60% aminoacid identity)to an expressed protein in rice (LOC_Os01 g68840), which appears in the MSU rice community annotation as a small, cysteine-rich protein named *CRP6*[40]. In previous work involving legumes [41], small cysteine rich peptides (CRPs) were classified as different classes of defensins, proteins that are active in conferring resistance to bacteria, fungi and viruses. The Cx(2)-CxCCx(6)-Cx(4)-CCx(4)-Cx(9)-Cx(6)-CxCx(2) (Figure 3b) arrangement of the cysteine domain in *TaCRP1*corresponds to the ma ternally expressed gene (MEG) family.

**Table 2:** Gene and EST analysis of the *ctg1034*'gene -island' genes

Gene	Position	Size (bp)	Number of exons	Number of amino acids	Wheat EST (>80% coverage)	E-value	Gene coverage	Wheat Unigene set
*TaEP1*	493556-496044	2489	2	551	CJ799192 (667 bp)	0.0	27% (over 1 exon)	Ta.11404
*TaCRP1*	500300-500735	436	2	118	BQ806045 (623 bp)	0.0	73% (over 2 exons)	Ta.14062
*TaZFN1*	502241-506766	4526	7	435	CK209603 (1147 bp)	7e-^144^	21% (over 5 exons)	Ta.11131

The last gene matched the NCBI non-redundant amino acid database was annotated as a zinc finger protein (*TaZFN1*). This gene was 4,525 bp, has a 7-exon gene structure and encodes a predicted protein of 430 amino acids (Table 2). The gene was shown to have high homology (82% identity) to the rice zinc finger CCCH domain-containing protein ZFN-like 2 (Q5JLB5) and well supported by wheat ESTs (Table 2) and a full-length wheat cDNA taken from the KOMUGI Wheat Genetic Resources Resource http://www.shigen.nig.ac.jp/wheat/komugi/. *TaZFN1 *was the only one of the four identified gene models that had a match to a full-length wheat cDNA; the cDNA match to the coding sequence of *TaZFN1 *was almost 100%, with only 2 base differences.

The NCBI Unigene EST profiles http://www.ncbi.nlm.nih.gov/unigene of each of the genes were compared in Table 3, showing the expression of each of the genes in wheat, barley (*Hordeum vulgare*) and rice tissue-specific EST pools. There was a low-level of transcription of the *EP1 *gene and no tissue specificity could be inferred from the data across all species. While there was a low-level of transcription found from the EST data for the *CRP1 *gene, there was clear seed-tissue specificity across all species relative to the expression in leaf and root tissues. The *ZFN1 *gene displayed much higher transcription levels than the previous two genes but like *EP1 *showed no tissue-specificity could be inferred.

**Table 3:** Wheat and Rice UniGene EST expression profiles of the three genes located within the *ctg1034 *gene island.

	UniGene Accession	Seed*	Leaf*	Root*	Stem*	Callus*
**EP1**						

Wheat	Ta.11404	**2 (21)**	**0**	**2 (11)**	**0**	**0**
	EST pool sizes	161877	57503	166795	93580	10594
Barley**	Hv.27853	**0**	**1 (17)**	**0**	**0**	**0**
	EST pool sizes	88535	111884	32853	65681	16046
Rice	Os.42759	**0**	**1 (5)**	**0**	**3 (23)**	**1 (6)**
	EST pool sizes	32419	171897	68247	126907	164917
						
**CRP1**						
Wheat	Ta.14062	4 (24)	0	0	0	0
Barley	Hv.19960	1 (11)	0	0	1 (15)	1 (62)
Rice	Os.65102	3 (92)	0	0	0	8 (48)
						
**ZFN1**						
Wheat	Ta.11131	34 (210)	8 (139)	6 (35)	11 (117)	1 (94)
Barley	Hv.8311	4 (45)	11 (98)	2 (60)	2 (30)	0
Rice	Os.19086	3 (92)	5 (29)	0	3 (23)	12 (2)

*The number of tissue-specific ESTs is shown and the extrapolated transcripts per million (TPM) are shown in brackets

**Barley EST profiles are also shown on the basis of BLASTN homology searches against the NCBI EST dataset.

### Comparative analysis using the gene-island-coding sequences from cereal genomes

The three genes found as a small cluster or island within the wheat *ctg1034 *region of chromosome 3B were used to search against the rice (*O. sativa ssp. japonica*) and *Sorghum bicolor *genome sequences and each genome was found to be syntenic over the region, showing both conserved gene order and orientation. The rice MSU version 6 annotation of chromosome 1 between 39,898,000 to 40,011,000 bp (Table 2) in the rice genome was used along with the *Sorghum *Sbi1.4 annotation models (from MIPS/PASA on v1.0 assembly preliminary Genome-scan annotation of assembly sbi0 http://www.phytozome.net) from chromosome 3 of *Sorghum bicolor *from between 71,051,000 and 71,068,000 bp (17 kb region).

The three genes from *ctg1034 *were also searched against the Brachypodium (*Brachypodium distachyon*) line Bd21 and maize (*Zea mays cv B73*) genome sequences using BLASTN and the top hits of all three were once again found to have conserved gene order and orientation. The equivalent of the wheat 16 kb region in the draft Brachypodium super13 scaffold sequence (1311-1327 kb) contained the conserved colinear genes and was annotated using the same protocol used to annotate the *ctg1034 *wheat genome sequence. The three genes were named *BdEP1*, *BdCRP1 *and *BdZFN1 *based on their rice protein matches in the MSU version 6 annotations (same as the wheat gene annotation, Table 4). A small genomic sequence gap within the draft sequence (4× sequence coverage) located inside exon 6 and intron 5 of *BdZFN1 *was filled on request to the Brachypodium genome-sequencing project (Bevan, M., pers. comm.) and reduced the size of the syntenic gene island sequence to 14 kb. A syntenic region containing the *TaEP1*, *TaCRP1 *and *TaZFN1 *genes in maize was located within the ctg131 FPC contig on maize chromosome 3. The maize genomic BAC clone containing all three genes (AC217295.3) was accessed from the maize genome sequence project website http://maizesequence.org/ and were annotated for all genes (Additional file 1) based on alignments to *Sorghum bicolor *gene annotations http://www.phytozome.net/sorghum with intron-exon structures confirmed using NCBI maize ESTs. The three genes were named *ZmEP1*, *ZmCRP1 *and *ZmZFN1*. Transposable elements were annotated using BLASTN against the TIGR Zea repeat database [42](Figure S2 in Additional file 2).

**Table 4:** Characteristics of three wheat genes identified to be syntenic and colinear genome sequences to the rice, Brachypodium and *S. bicolor *genome sequences.

		Rice Genome		*S. bicolor *Genome		Maize Genome		Brachypodium Genome	
**Gene**	**Description**	**Gene Match**	**Chr1 position (strand)**	**Gene Match**	**Chr3 position (strand)**	Gene Match*	**AC217295.3 Position (strand)**	Gene Match*	**Super_13 position (strand)**

*TaEP1*	Expressed Protein	LOC_Os01 g68830	39991137-39993669 (+)	Sb03 g043790	71051976-71055103 (+)	*ZmEP1*	65318-67814 (+)	*BdEP1*	1312106-1314592 (+)
*TaCRP1*	Expressed Protein	LOC_Os01 g68840	39997846-39997379(-)	Sb03 g043800	71059536-71060101(-)	*ZmCRP1*	117899-118353 (-)	*BdCRP1*	1317872-1318293 (-)
*TaZFN1*	Putative zinc finger CCCH type domain- containing protein	LOC_Os01 g68860	40009733-40005685 (-)	Sb03 g043810	71062164-71067135 (-)	*ZmZFN1*	119558-125029 (-)	*BdZFN1*	1319767-1324280 (-)

*denotes the Brachypodium and maize genes that were annotated along with wheat in this study.

*TaEP1 *showed the highest level of amino-acid conservation (Figure 3a) among the *EP1 *genes in the cereal genomes. The highest similarity was with Brachypodium *BdEP1 *(88% identity at the amino acid level), followed by rice LOC_Os01 g68830 (85%), *S. bicolor *Sb03 g043790 and maize *ZmEP1 *(both 81% identity). *TaEP1 *also had 49% amino acid identity to a peptidase protein AT3G07210 in Arabidopsis. The Brachypodium *BdEP1 *also had a good conservation of non-coding nucleotide sequence within the intron of *TaEP1*. The intron sequence of *BdEP1 *was 70% identical over 836 nucleotides with 85% sequence coverage compared to the rice comparison, which had only 16% coverage over the 826 nucleotides. There was a small region of 110 bp near the start of the intron that did not show any homology. The intron sequence of maize *ZmEP1 *compared to *S. bicolor *Sb03 g043790 was also highly conserved (78% identical over 821 nucleotides with 99% coverage).

The large 7-exon *TaZFN1 *gene, much like *TaEP1*, showed significant homology to its Brachypodium orthologous sequence. The amino acid sequences of both genes showed 89% identity over 387 amino acids, with only a small section located within the first exon lacking homology. Both *S. bicolor *(403 amino acids) and rice (443 amino acids) showed 82% identity compared to *TaZFN1*, with maize showing the least similarity at amino acid level (77% over 429 amino acids). The amino acids from the first exon (1-80 amino acids in Figure 3c) seemed to contain the most sequence divergence when compared to the rest of the genome sequences. As was found in *TaEP1*, there was a significant conservation of the non-coding sequence between *TaZFN1 *and *BdZFN1*, especially in introns 3, 5 and 6 which all have a nucleotide sequence coverage of >62%. The *ZFN1 *gene sequence between maize and *S. bicolor *was also well conserved (84% identity over 4915 nucleotides) except for two small inserts contained within the fifth intron of *ZmZFN1*, one insert of 1,166 bp and another of 118 bp. The larger 1,166 bp insert contained two small transposable elements *CASINE *(63 bp) and *F524 *(91 bp) classified as SINE elements from the non-LTR retrotransposable element group.

By comparison, the *TaCRP1 *showed the least amount of homology across the genomes when analysed at the amino acid level (Figure 3b) relative to *TaEP1 *and *TaZFN1*. Once again, Brachypodium had the highest amino acid homology (85% identity), followed by rice (66%), *S. bicolor *(62%) and maize (60%). The low amino acid identity was due to insertions within the rice, maize and *S. bicolor *amino acid sequences. *S. bicolorSb03 g043800 *had a 33 amino acid insertion located just after the signal peptide and before the cysteine domain. It is also the only *CRP1 *sequence out of the five syntenic gene copies that had only one exon (no intron). The cysteine domain located in the last 50 amino acids of the gene was conserved in all genomes (~75% identity). The only significant difference in the cysteine-rich domain across all genomes was a six amino acid insertion (all glycine residues) within the rice LOC_Os01 g68840 sequence. Exon 1 in all sequences that contained multiple exons showed little nucleotide and amino acid sequence homology and exon 2 was the only region found to have significant sequence conservation. Exon 2 contained the coding sequence of the cysteine-rich domain. No significant intron sequence similarity could be identified across the genomes.

### The syntenic gene island region across five genomes

The *ctg1034 *14 kb gene island was analysed across the available genomic sequences including Arabidopsis genome sequence [43], the grapevine (*Vitis vinifera*) genome sequence [44] and the wheat, Brachypodium, *S. bicolor*, rice and maize genome sequences. The 14 kb, three-gene region containing *TaEP1*, *TaCRP1 *and *TaZFN1 *did not show any conservation of colinearity in the Arabidopsis genome sequence. Chromosome 4 of the grapevine genome did have homologs of *TaCRP1 *and *TaZFN1 *located within a 17 kb window (in the same relative orientation as in wheat) but also contained a small (65 predicted amino acid) gene found in between. This small gene coding for a 65 aa protein has no rice or Arabidopsis BLASTX hit and could possibly be within a transposable element.

**Figure 4:**
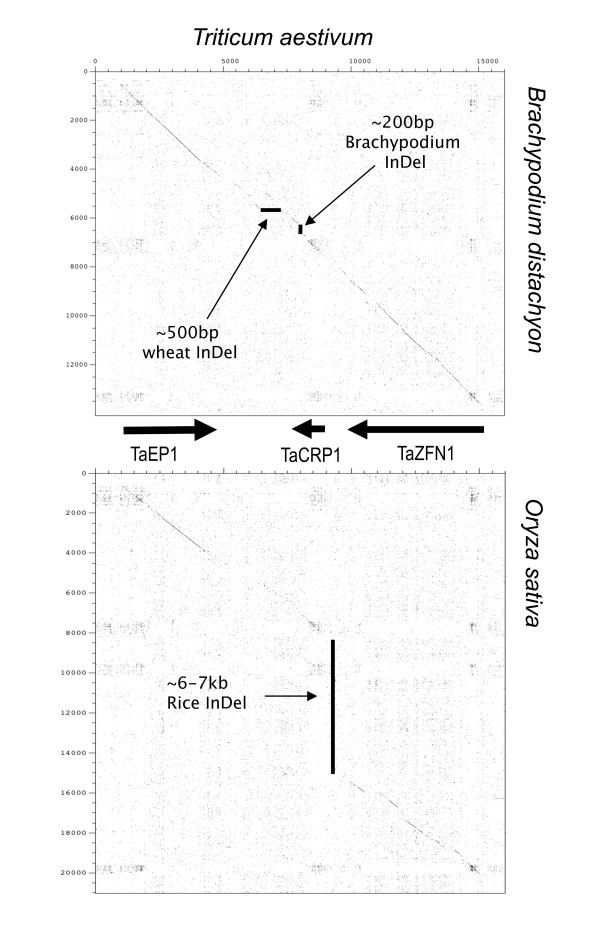
**Pair-wise sequence comparison of wheat and Brachypodium gene island regions against the wheat and rice gene island regions**. The wheat genes (*TaEP1*, *TaCRP1 *and *TaZFN1*) are annotated on the figure and the arrows indicate the proposed insertion-deletion (InDel) events resulting in sequence movement between the species. Wheat contained a ~500 bp InDel, while Brachypodium and rice showed ~200 bp and ~6-7 kb InDels respectively.

There are large size differences of the gene-island regions between wheat (16 kb) and both maize (63 kb) and rice (22 kb) resulted from an insertion-deletion event (InDel) in both cases. Comparing the genome sequences of rice against the wheat gene island region shows a 6-7 kb sequence variation (InDel) between the *OsCRP1 *and *OsZFN1 *genes (Figure 4). The proposed InDel within the rice genome contains a hypothetical protein annotation LOC_Os01 g68850 and contained many MITE and DNA transposons found in the TIGR Oryza Repeat Database v3.1 (repeat_212948; 230577; 212949; 205504; 216962) including a MULE or Mutator-like element (BPM2_1011). The LOC_Os01 g68850 open-reading frame was identified using FGENESH and contained a high amount of repetitive elements. Furthermore, the match to sequences in the EST databases of rice indicated the largest region of significant homology (94% identity to rice EST CK043734) was only 208 bp and thus it seems unlikely to be an active entity.

**Figure 5:**
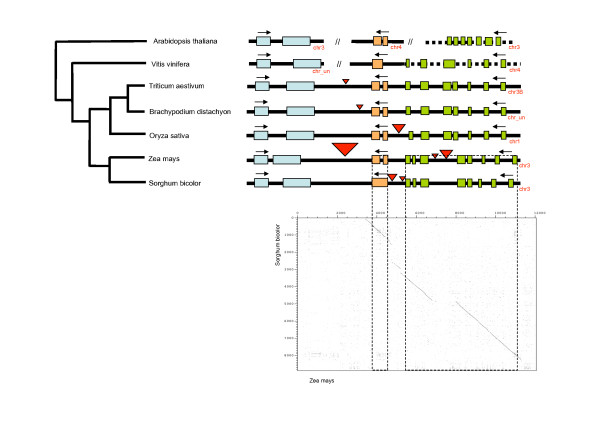
**A gene island sequence summary figure next to a deduced evolutionary tree from analysed genome sequences homologous to the wheat chromosome 3BL *ctg1034 *gene island**. The red triangles indicate proposed insertion-deletion (InDel) events identified in this study. An example of a dot plot indicating the occurrence of InDels is shown beneath the pair-wise sequence comparison between the maize and *S. bicolor*. The analysis demonstrates the location of the InDels between the start of the cysteine-rich peptide gene (*CRP1*) to the end of the Zinc finger protein (*ZFN1*) region. A detailed analysis of the differences in length in this region between the genomes analysed (red triangles) is provided in the Additional file 2.

Overall, in five of the genome sequences used in this study, there was a highly conserved gene sequence, order and orientation, when compared to the wheat *ctg1034 *gene island (Figure 5), even though the size of the region differed considerably; greatest in the maize genome (63 kb), followed by rice (22 kb), *S. bicolor *(17 kb) and Brachypodium (14 kb) (Figure 5). The 4× draft Brachypodium super13 scaffold sequence showed the highest nucleotide coverage (53% coverage), followed by rice (31%), *S. bicolor *(29%) and maize (23%) when each sequence was compared to the 16 kb gene island wheat genomic sequence using NCBI BLAST 2 Sequences (BLASTN) [45]. A detailed analysis of the differences in length in this region between the genomes analysed (Figure 4 and the red triangles in Figure 5) is provided in the Additional file 2.

## Discussion

In this study a group of twelve hexaploid wheat (*Triticum aestivum *cv Chinese Spring) BAC clones from chromosome 3B were assembled into a 783,553 bp genomic sequence (called *ctg1034*) that was then analysed for biological features.

### Diverse gene functions in a conserved gene-island

The three gene-coding sequences on *ctg1034 *(*TaEP1*, *TaCRP1 *and *TaZFN1*), located within a 14 kb sequence, all show different sequence characteristics and a range of expression patterns and functions based on rice annotations. *TaEP1 *is highly conserved in plant genomes (even in Arabidopsis) but does not show any distinct characterised function in sequenced genomes such as *S. bicolor *and rice. *TaCRP1*, on the other hand, is a small cysteine-rich protein, a protein from a diverse, well characterised protein family that is involved in pathogen defence [40]. *TaZFN1 *was like *TaCRP1*, a well-characterised protein from a large family of transcription factors that have been extensively studied. Furthermore multiple full-length cDNA sequences of *TaZFN1 *in rice indicate alternative splicing forms of the gene product (LOC_Os01 g68860.1, LOC_Os01 g68860.2 and LOC_Os01 g68860.3) exist. EST expression profiles from 3 did not provide any clear indication of co-expression of the three genes; *TaZFN1 *was striking in its relatively high levels of expression in a range of tissue compared to *TaEP1 *and *TaCRP1*. This is consistent with the genes being involved in independent pathways within cells.

### Gene island structure is maintained over the evolution of plant genomes despite the occurrence of InDels

Conservation of colinearity between grass species has been reported extensively at a micro-level [17,46] but even in colinear regions, gene order and orientation may not be conserved [6,9,17,46-48]. The conserved gene-islands described between wheat and other major crop species such as rice [11], have been limited because of the use of EST and single BAC sequences (unlike the large assembled sequences created in this study), with the associated lack resolution making it difficult to reach definitive conclusions about the level of conservation. While conserved gene islands at gene-rich orthologous loci have been shown, small genetic rearrangements were found to dramatically change gene order in maize and *S. bicolor *[48] and a mosaic of rearrangements between rice and wheat were also reported, with the conservation of gene orientation only limited to local duplications in one species [49].

The gene island provides a contrast to the concepts developing from mammalian genome studies that repeat sequence regions may be hot-spots for structural change in gene regions [50]. While the relative order and orientation of the genes within the *ctg1034 *gene island has been maintained, the intergenic region was not immune to changes during the course of its evolution. Figure 5 shows a summary evolutionary tree deduced from colinearity and sequence similarity seen in this study over the *ctg1034 *gene island. This evolutionary tree is consistent with taxonomic relationships [51].

Despite the large amount of repetitive elements that surround the island, only the rice and maize genome (Figure 5 and Additional file 2) sequences showed post-divergence TE insertions. An insert within maize is not unusual, as [52] has shown that even maize inbred lines can show intra-specific variation in genetic colinearity. High sequence diversity in maize can be caused by TEs such as *Helitrons *[52-54]. The 6-7 kb rice insertion mostly contained miniature inverted TEs (MITEs), known to insert into and be associated with gene-containing regions [23,55,56] and [36] suggested that these small TEs might also have a role in modification of expression in neighbouring genes. Another TE found within the insert was a *MULE *transposon. So called *Pack-MULEs *are extremely common in the rice genome [57] and have been implicated in the evolution of genes within higher plant genomes through their ability to capture and carry fragments of genomic DNA to create new open reading frames.

### Gene island location on chromosome 3BL

The gene density in this study was identified to be quite low at 1 gene per 260 kb. In contrast, gene densities in *Triticeae *genomes (most notably barley and wheat) have been reported to be much higher (1 gene per 9 kb [58] and 1 gene per 4-5 kb [19]), possibly reflecting the initial targeting of important agronomic genes in distal regions of chromosomes [59] rather than a random representation of the genome sequence. Random wheat BAC sequencing [7] indicated that even in gene-rich regions of wheat, the gene density is considerably lower than previously thought (approximately 1 gene per 75 kb). Genetic mapping studies showed Sc3-119 and Sc3-120 ISBP markers from *ctg1034 *were located within the terminal deletion bin 3BL-7 on chromosome 3B (Figure 2), a deletion bin known to contain two major C-bands [59]. Previous cytogenetic studies of the wheat chromosomes suggested a large variation of gene-density over the entire length of chromosomes [60,61] and it is feasible that particularly low gene densities occur within C band regions. The low gene density found in this study is more consistent with studies in *Triticeae *genomes of more proximal regions of chromosome arms (1 gene per 518 kb [62]) and regions of low recombination (1 gene per 175 kb [63]).

The two representatives of tandem repeat families located within the first 75 kb of the *ctg1034 *sequence also indicate a relationship to heterochromatin, with one (AY249982) being previously identified to be a D-genome centromeric heterochromatin tandem repeat sequence [35,36]. The presence of a representative of this D-genome specific repeat in the chromosome 3B sequence suggest specific amplification of the sequence family must have occurred in the D genome even though single copy representatives were located elsewhere. The presence of representatives of sequences normally located as tandem arrays in heterochromatin and the presence of high *gypsy *LTR content support our proposal that this *ctg1034 *genomic sequence is located within a C-banding region in deletion bin 7 of chromosome 3BL.

The highly repetitive element content in *ctg1034 *(81.81%) was not unusual in wheat sequences, as studies have shown repetitive element contents of greater than 70% [21,56,64], however the high content of the *gypsy-like *LTR retrotransposon is significant in this study (>50% of the entire contig). A previously sequenced wheat BAC clone from the same chromosome 3BL-7 deletion bin [5] also found a similar high proportion of *gypsy-like *LTR-retrotransposons. In the present study, chromodomains were shown to be located within the internal polyprotein domain of two *gypsy*-LTR retrotransposons, and are thus potentially key components defining the condensed nature of heterochromatin [29,65]. One of the potential chromodomains (*Latidu *LTR *gypsy *retrotransposon) was located immediately adjacent to the gene island in *ctg1034*.

The unusual properties of heterochromatin that would contribute to the unique conservation of both the gene order and relative gene orientation found in this study include: (a) the low levels of recombination and gene activity associated with heterochromatin [33,66]; (b) the high level of transcript processing by the RNAi machinery that mediates chromatin structure [67-71]. These properties could contribute to the relative isolation of the gene island from the normal processes that lead to genome rearrangements.

## Conclusion

BAC shotgun sequencing of the FPC physical map contig *ctg1034 *from the genome of chromosome 3B has provided the largest assembly of a wheat genome region to date (783,553 bp). Annotation and comparative analysis with four other plant genomes of (*B. distachyon*, *O. sativa*, *S. bicolor *and *Zea mays*) identified an island of three genes showing complete conservation of gene order and orientation. This conservation could not be readily accounted for by shared functions between the genes and it is proposed that *ctg1034 *is located within a heterochromatic C-band region of deletion bin 3BL7 based on the identification of representatives of heterochromatin tandem repeats and presence of significant matches to chromodomain-containing *gypsy *LTR retrotransposable elements. We speculate that this location, among other highly repetitive sequences, may account for the relative stability of the gene island through insulating the gene island from normal recombination processes.

## Methods

### Mapping of selected BAC clones

Selected clones were mapped to chromosome 3B using insertion site based polymorphism (ISBP) markers [7,23]. Two ISBPs (Sc3-119 and Sc3-120) from *ctg1034 *were located on the long arm of chromosome 3B using standard deletion stocks of wheat [2]. The Sc3-119 primer 5'-TCCAAGACGTTTCTTCCACC-3' and 5'-GAGGTGACGTGGCATCATTA-3' generated a 158 bp product and was located at 510,455 bp within the *ctg1034 *sequence. The second ISBP used, Sc3-120 with primer '5-GCCCCTGGCTTGTATTATGA-3' and 5'-TCAGCTGAAGGGTCGTTTTT-3' giving a 190 bp specific fragment located at 523,485 bp. The PCRs carried out on genome DNA from the standard 3B deletion stocks and the 162 double haploid lines from the cross Cranbook × Halberd followed standard reaction conditions [25,72].

### BAC shotgun sequencing

*E. coli*-freed DNA from BAC clones was extracted with Qiagen Large-Construct Kit (QIAGEN, Cat. No. 20021) and mechanically sheared with HydroShear as recommended by ABI applied biosystems https://products.appliedbiosystems.com/ab/en/US/adirect/ab?cmd=catNavigate2&catID = 604432, generating a concentrated smear ~3-5 kb in length. The sheared fragments were blunt ended with mung bean nuclease and dephosphorylated with Shrimp Alkaline Phosphatase (SAP). The short fragments were then tailed with A by PCR using standard procedures.. Fragments ranging from 3-5 kb in size were isolated and ligated into a pCR4-TOPO vector and transformed into TOP10 electrocompetent cells (Invitrogen, Cat. No. K4580-01). The clones were sequenced from both directions with T3 and T7 primers using BigDye3.1 termination chemistry and run on an ABI Prism 3730 XL capillary sequencer (Applied Biosystems, Foster City, Calif., USA). Base calling, quality assessment and sequence assembly were done using the PHRED/PHRAP software package [23]. Gaps were filled by primer walking using adding dGTP mix and DMSO in the sequencing reaction system.

### Sequence Analysis and Annotation

Repetitive DNA analysis was carried out using Repeatmasker (Smit et al. 1996-2004, http://www.repeatmasker.org) and BLAST local alignment searching [26] against the Triticeae repetitive element (TREP) database http://wheat.pw.usda.gov/ITMI/Repeats/ and the *gypsy *mobile elements database (GyDB) [34]. Open reading frames were identified by the use of FGENESH http://www.softberry.com/, GENSCAN [73] and GlimmerHMM [37]. Sequence homology searching was carried out by BLAST programs with a cut-off E-value of 1e^-30 ^for nucleotide sequences and 1e^-10 ^for amino acid sequences. All EST matches to genes were required to have >80% coverage over the EST sequence. Protein domains were identified by searching the Pfam protein family database [74], as well as the conserved domains database (CDD) at NCBI [75]. InterProScan [76] was run against the InterPro protein domain database [77], which also includes a signal peptide and Trans-membrane search. Pair-wise sequence comparisons were carried out using DOTTER [27] and multiple sequence comparisons were carried out using the CLUSTALX [78]. The hidden markov model search program HMMer [30] was also used. Graphical display of the sequence map was produced with WICKERsoft™ scripts.

### Comparative Sequence analysis

Genome sequence annotations used in this study include release 5 of the TIGR rice pseudomolecules [38], the 4× draft genome sequence of *B.distachyon *http://www.brachypodium.org/, the 2a.50 release of the Maize (*Z. mays*) genome sequence http://www.maizesequence.org and release 1 of the *S. bicolor *genome http://www.phytozome.net/sorghum. Multipipmaker [79] and Multidotter [80] were used for nucleotide sequence comparisons. CLUSTALX was used to compare amino acid sequences and Multidotter, Mauve [81] and ACT [82] was used to compare multiple genome sequences at one time.

## Authors' contributions

All authors read and approved the final manuscript.

JB, TW, MB, RAs: Assembly of the genome sequence and annotation of genes and TEs

EP, CF: Cloning and assembly of BACs from chromosome 3B

XK, JZ: Sequencing of BAC clones

WM: Mapping of ISBPs to wheat genetic map

## Supplementary Material

Additional file 1 **Supplementary Table S1**. Gene Annotation of Maize Chromosome 3 BAC AC217295.3. Click here for file

Additional file 2 **Supplementary INDELs file**. Detailed of the InDels shown as red triangles in Figures 4 and 5 of the main manuscript. Click here for file
