# Supplementary material for: A highly conserved gene island of three genes on chromosome 3B of hexaploid wheat: diverse gene function and genomic structure maintained in a tightly linked block
Source: BMC Plant Biol. 2010 May 27;10:98. doi: 10.1186/1471-2229-10-98 (PMC3017796; doi:10.1186/1471-2229-10-98)
Supplement: Additional file 1 — Supplementary Table S1. Gene Annotation of Maize Chromosome 3 BAC AC217295.3. [file 1471-2229-10-98-S1.PDF]

Supplementary Table 1: Gene Annotation of Maize Chromosome 3 BAC AC217295.3.

						**Protein Analysis**		**Maize EST Analysis**		
**Gene**	**Position**	**Size (bp)**	**Number of exons**	**Number of amino acids**	**Syntenic Sorghum gene match**	**BLASTx Top Hit (Swissprot)**	**Eval**	**Maize EST**	**Eval**	**Maize Unigene set**
ZmPK1	22656-26534	3879	1	1293	Sb03g043820	NP_001045202 (Os01g0917500)	0.0	DR803269	0.0	Zm.23212
ZmUK1	31961-32341	655	2	177	Sb03g043840	No significant Hit	-	DR960750	0.0	Zm.40881
ZmZFNR1	40168-41085	918	1	306	Sb03g043850	NP_001045204 (Os01g0917900)	7e-65	DR958212	0.0	Zm.64787
ZmEP_1	65318-67814	2497	2	551	Sb03g043790	NP_001045199 (Os01g0917200)	0.0	EE183402	0.0	Zm.36208
ZmEP_2	117899-118353	455	2	122	Sb03g043800	ACF86199 (unknown maize)	3e-42	EE292907	0.0	Zm.41612
ZmZFN1	119558-125029	5472	7	429	Sb03g043810	ACF82040 (unknown maize)	0.0	EE039211	0.0	Zm.87717
