# Supplementary material for: A highly conserved gene island of three genes on chromosome 3B of hexaploid wheat: diverse gene function and genomic structure maintained in a tightly linked block
Source: BMC Plant Biol. 2010 May 27;10:98. doi: 10.1186/1471-2229-10-98 (PMC3017796; doi:10.1186/1471-2229-10-98)
Supplement: Additional file 2 — Supplementary INDELs file. Detailed of the InDels shown as red triangles in Figures 4 and 5 of the main manuscript. [file 1471-2229-10-98-S2.PDF]

Detailed of the InDels shown as red triangles in Figures 4 and 5.

The analysis of the rice InDel identified multiple high homology matches (all with E-values < $1e^{-80}$) with rice ESTs in at least four different locations within the rice InDel but all matches were not unique to the particular location and were located on multiple rice chromosomes confirming their repetitive nature. No gene models, other than the repeat-containing LOC_Os01g68850 rice gene mentioned above, were discovered in the InDel region (Figure S1).

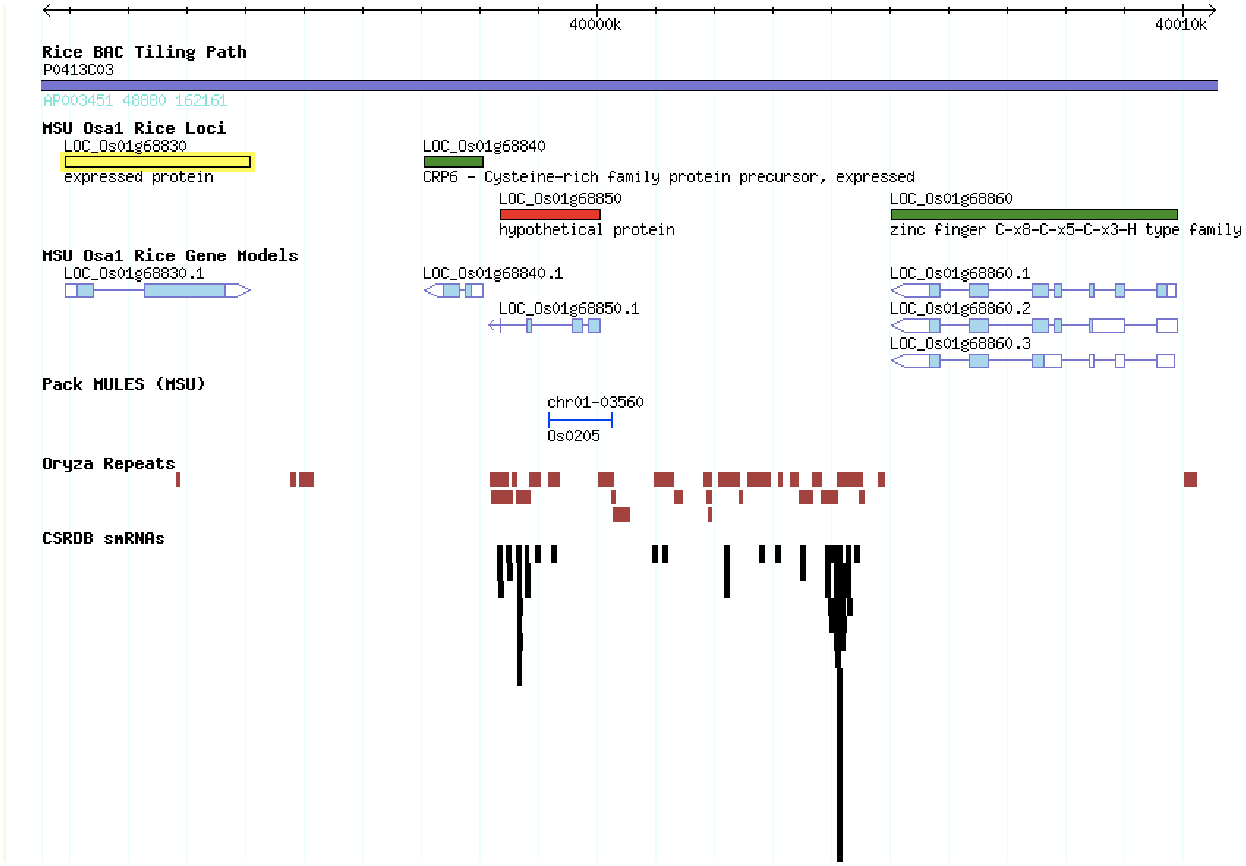

Figure S1. Detailed analysis of the rice InDel region shown in Figures 4 and 5 of the main manuscript. The InDel is located between the CRP6 and Zinc finger genes (in green) and showed a strikingly asymmetrical distribution of snRNA's (lowest row of analyses in black)

This region was also analysed in rice for the presence of small RNAs (smRNAs) such as miRNAs, siRNAs and trans-acting siRNAs (ta-siRNAs), from the Cereals Small RNA database (CSRDB). 24 smRNAs (between 18-23mer smRNAs) were located within the rice insert region, 23 of which were located at the 5' and 3' boundaries of the inserted region. A further 35, 24mer-smRNAs were also located within this region, once again the majority of which located along the terminal ends of the inserted region (Figure S1).

Unlike the proposed rice InDel identified above, the proposed maize InDel region (main manuscript Figure 5) is located between Zm*EP1* and Zm*CRP1* (Zm*EP2*). The area contains a high proportion of transposable elements (Figure S2), with the majority of the insertion (38,778bp or 77.4% of the insertion sequence) being made up of the *gypsy*-like LTR retrotransposable element called *Huck*. The *Huck* family of LTR retrotransposons are abundant among maize genomic sequences.

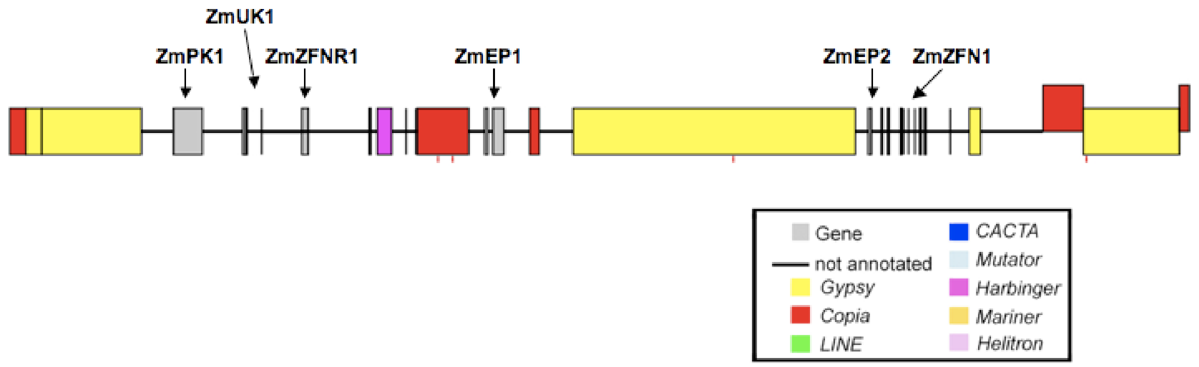

Figure S2. Detailed analysis of the maize InDel region shown in Figures 4 and 5 of the main manuscript. In contrast to the rice InDel, the region between ZmEP1 and ZmEP2was composed mainly of retrotransposable elements (color coded according to the inset provided).

Further comparative sequence analysis between other colinear genome sequences in the gene island (wheat and Brachypodium) revealed a number of smaller insertions (main manuscript Figure 4). Two Indels in wheat (~600bp) and Brachypodium (~300bp) shown in the main manuscript Figure 4 were found between the syntenic EP1 and CRP1 genes but were shown to not contain matches to any transposable elements in the Triticeae repeat sequence database (TREP). Two small insertions (similar to the two insertions found within intron 5 of *ZmZFN1*) were also found within *S. bicolor* (main manuscript Figure 5) between *SbCRP1* and *SbZFN1* by comparing the maize and *Sorghum* genome sequences, with one insert being 1,044bp and the other 247bp. Within the larger 1,044bp insert there was a significant 444bp hit (E-value of $3e^{-55}$) to the *S. bicolor* ‘Candystrip1’ Transposable element of the TIGR Plant repeats database (http://plantrepeats.plantbiology.msu.edu/index.html).
